# Rapid Generation of Fusable Cell Beads for Multi‐Scale Human Living Materials Assembly

**DOI:** 10.1002/smtd.202501450

**Published:** 2026-01-15

**Authors:** Beatriz S. Moura, Maria V. Monteiro, Joana F. Soeiro, Nuno J. O. Silva, Vítor M. Gaspar, João F. Mano

**Affiliations:** ^1^ CICECO‐Aveiro Institute of Materials Department of Chemistry University of Aveiro Campus Universitário de Santiago Aveiro Portugal; ^2^ Cellularis Biomodels Pci Creative Science Park Aveiro Region Ílhavo Portugal; ^3^ CICECO‐Aveiro Institute of Materials Department of Physics University of Aveiro Campus Universitário de Santiago Aveiro Portugal

**Keywords:** bottom‐up assembly, cellgel beads, hierarchical constructs, living materials, metabolic glycoengineering

## Abstract

Three‐dimensional self‐assembled cellular aggregates, such as spheroids, provide unique building blocks for bottom‐up tissue engineering and in vitro disease modeling. Nevertheless, traditional spheroid production methods require prolonged cell aggregation times and are highly dependent on cell type, requiring frequent optimization steps. Additionally, spheroids’ size is dependent on their cell density, preventing a control over their final volume. Herein, a methodology combining metabolic glycoengineering and click chemistry with superhydrophobic surfaces is described to rapidly create spherically structured living bead units, that can surpass the fabrication constraints of conventional spheroids. Compared to spheroids produced in low attachment settings, the living beads comprising various cell types (i.e., stem, endothelial, and cancer cells) are rapidly produced and demonstrate enhanced cell viability and cell spreading over 14 days, while maintaining principal spheroid characteristics, namely the fusion into multi‐scale living materials and cellular migration capabilities. In addition, this methodology enables the production of living beads with controlled size, independently of cell density, overcoming a key limitation of current spheroid production methods. The enhanced reproducibility, reduced cell assembly time, and improved handling make these spherically structured living beads a valuable alternative, with broad application in bottom‐up tissue engineering approaches and disease modeling applications.

## Graphical abstract

Living beads are produced through the combination of metabolic glycoengineering, click chemistry, and superhydrophobic surfaces, to develop a spheroid alternative for bottom‐up tissue engineering and disease modeling. The living beads present a size independent of cell density and a rapid fabrication methodology, providing a platform that can fuse into multi‐scale structures or be integrated into other tissue‐mimetic platforms.

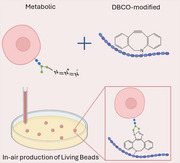

## 1 Introduction

The fabrication of 3D cell assemblies has major advantages for tissue engineering and disease modeling applications. The better representation of in vivo tissues using these culture systems relies on the fact that they provide closer cell–cell and cell‐matrix interactions, increased production of matrix, and a better representation of the nutrient and oxygen gradients, when compared to their corresponding 2D cultures [1, 2]. The three‐dimensionality provides complexity that results in closer cell proliferation gradients and cell migration profiles to those found in vivo, as well as mimicking the chemical and mechanical microenvironment [3].

Among the available physiologically relevant cell assemblies, 3D spheroids provide an excellent cell‐rich model since they yield a biomimetic structure, that resembles the 3D architecture of tissues and matrix deposition [4] and is extensively used in cancer, considering they mimic the 3D nature of tumor, replicating their microstructure and cell–cell contacts [5, 6, 7].

Whereas spheroids have been key in advancing tissue engineering and drug screening in a more accurate representation of the in vivo environment, they still come with limitations. The current manufacturing of spheroids requires long cell aggregation times, which are highly dependent on cell type and chosen fabrication method [8]. Additionally, spheroids exhibit a size dependent on the number of cells they contain [9], which result in variations in cellular behavior and the microenvironment, with increased cellular senescence for larger spheroids of mesenchymal stromal cells, as well as changes in their secretome composition being reported [10]. Spheroids with uniform size, regardless of initial cell number, as well as improved production speed, would benefit greatly the studies using these platforms, by providing improved cell–cell interactions [11], and improving experimental reproducibility. Accordingly, addressing the current limitations in size uniformity and production speed will require innovative approaches, and cell surface modification toolboxes offer a promising solution. Such approaches enable precise regulation of cellular coupling and spheroids’ formation in chemistry‐controlled time scales, compatible with biological processes [12]. Cell surface functionalization via metabolic glycoengineering is a powerful approach that incorporates chemically modified sugar analogs into the cell surface, enabling their precise manipulation [12, 13]. This technique facilitates control over cellular interactions and assembly, promoting the creation of densely packed cell‐based platforms [14, 15]. Previous work reported the development of Cellgels, cell‐rich structures with tissue‐like characteristics, produced through the combination of metabolic glycoengineered cells and a hyaluronan tethering biopolymer [15]. The tissue biomimetic characteristics of the Cellgel technology can be harnessed to develop platforms that enhance existing spheroid production strategies, by integrating Cellgels with complementary technologies, such as superhydrophobic surfaces.

By taking inspiration from nature, superhydrophobic surfaces have been engineered to repel water, and therefore being used to fabricate round objects [16], whether composed solely of biomaterials [17, 18] or with cell‐laden materials [19, 20]. These surfaces have been used to encapsulate cells and biomolecules, since its chemistry creates low surface energy that will induce the creation of a nearly spherical shape of a dispensing liquid volume containing the cargo, which offers an improved encapsulation efficiency.

In this work, the metabolic glycoengineering of cells together with the use of superhydrophobic surfaces is employed as a methodology to rapidly produce spherically shaped living constructs that exhibit relevant cell viability and physiomimetic cell morphology. Using this technology, we hypothesize that it is possible to obtain fusable units, with the possibility to decouple cell number and object size. We also show the versatility of this technology by exploring several cell types, including stem cells, endothelial cells, and breast cancer cells, or the possibility of placing these units within other cell‐dense platforms.

Overall, we hypothesize that, by combining these technologies, it is possible to develop a feasible alternative for the current use of spheroids, especially for the bottom‐up tissue engineering and drug discovery fields.

## 2 Results and Discussion

Spheroids have been fundamental living building blocks in tissue engineering and disease modeling, providing a highly valuable platform for drug testing, in the latter [21]. However, they present diverse limitations, namely restricted complexity, as obtaining reproducible organization with multiple cell types within a spheroid can be challenging [22]. Additionally, oxygen and nutrient gradients are often present, due to their 3D, mostly very compact organization that impede diffusion. Maintaining spheroids with uniform cell density can be challenging, as their dimensions are often dependent on the chosen method of spheroid formation, impacting the reproducibility and precision [23]. Moreover, spheroids exhibit a size dependency on initial cell number, which directly influences the resulting cell density within the structure. This further hinders the reproducibility and efficacy in drug testing. Developing spheroid‐like structures with a volume independent of cell density would improve experimental control, enabling the study of the effect of cell density, by controlling the space between cells and consequently mass transfer within the structure. Such platform could improve reproducibility and provide more precise and informative studies on areas such as tissue engineering, cancer research, and drug testing.

### 2.1 Living Bead Unit Production and Size Characterization

**FIGURE 1:**
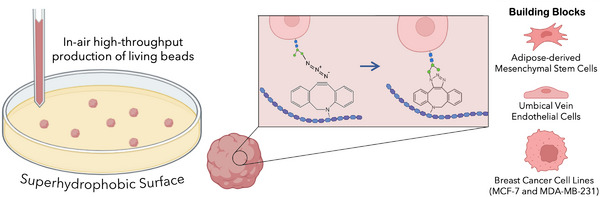
Schematic representation of in‐air production of living beads with superhydrophobic surfaces.

To establish living bead units, we have explored metabolic glycoengineering for installing azide groups at the cell's surface in combination with a hyaluronan biopolymer, a component of human extracellular matrix (ECM), modified with strained alkyne groups (i.e., Dibenzocyclooctyne (DBCO)) to generate the living beads via click chemistry. By using this approach together with superhydrophobic surfaces, we were able to rapidly fabricate spherically structured living bead units of different human cell types (Figure 1). These human cell types were selected as representatives of a range of biological behaviors, including stem and endothelial primary cells, to validate the methodology in stromal and vascular‐relevant scenarios, while the breast cancer cells enable the validation of the methodology with difficult‐to‐aggregate cells. Previous studies demonstrated the possibility of developing living materials with cell unit programmability and tissue‐like features, including cell density and self‐healing [15]. Since the cells themselves establish connections with the tethering biopolymer, cell density can be tuned, and living bead size can be controlled independently of the number of cells in their assembly. Therefore, we employed this technology and compared it to spheroids produced with the ultralow attachment plate methodology.

**FIGURE 2:**
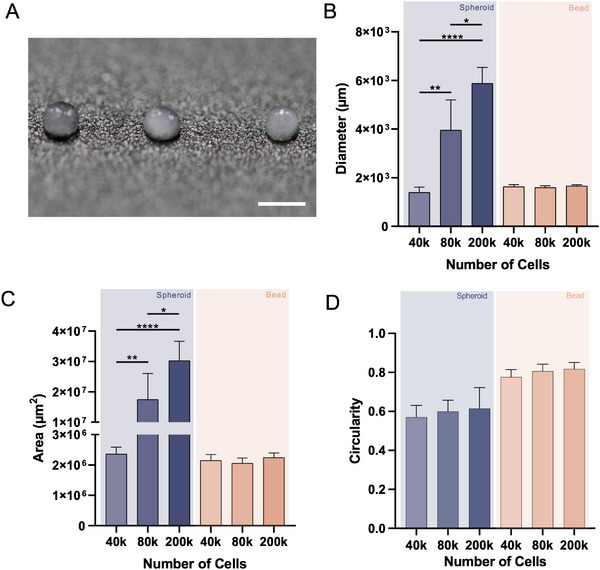
Development of mesenchymal stem cell living bead units and quantitative comparison with standard spheroids. (A) Micrograph of living beads with different cell densities (20, 40, and 100 million cells/cm^3^, from left to right), showcasing little differences in living beads’ size. Scale bar: 2 mm. (B) Spheroid and living bead diameter quantification, after 3.5 h of maturation and cross‐linking, respectively. Data presented as mean ± s.d (*n* = 9, 3 technical replicates from 3 biological replicates). (C) Spheroid and living bead area quantification, after 3.5 h of maturation and cross‐linking, respectively. Data presented as mean ± s.d (*n* = 9, 3 technical replicates from 3 biological replicates). (D) Spheroid and living bead circularity quantitative measurements, after 3.5 h of maturation and cross‐linking, respectively. (1 – perfect circle, 0 – line). Data presented as mean ± s.d (*n* = 9, 3 technical replicates from 3 biological replicates).

By combining the mentioned strategies, it was possible to establish living beads of mesenchymal stem cells with different cell densities, but similar sizes (Figure 2A). Analysis of optical contrast microscopy images was performed to compare the diameter, area, and circularity of both structures, after 3.5 h of assembly of living beads and at three different cell densities. Results show that spheroids have a diameter and area that are dependent on cell number, with spheroids of 4 × 10^4^ cells having an average diameter of 1397 µm and area of 2.4 × 10^6^ µm^2^, while spheroids of 2 × 10^5^ cells have an average diameter of 5878 µm and area of 3 × 10^7^ µm^2^. Using our technology, it was possible to produce living beads of three different cell densities, but with constant diameter and area values, all in the range of 1600 µm and 2.1 × 10^6^ µm^2^, respectively, showing an independence between size and cell number (Figure 2B,C). Additionally, it is possible to observe that the diameter and area of the structures show more consistency in the living beads compared to the spheroids, where the standard deviation is more pronounced. Concerning the circularity, the living beads provide an improved platform compared to the spheroids, with values ranging from 0.787 to 0.822, which are closer to 1 (perfect circle), compared to the values of spheroids, which range from 0.570 to 0.664 (Figure 2D). This is related not only to the fact that spheroids are formed by random self‐aggregation, but the aggregation process also takes longer than the living beads to be established, and therefore, more time is needed to get an improved shape and circularity. Our platform demonstrates enhanced assembly time and offers improved handleability when compared to spheroids.

### 2.2 Comparison of Established Living Beads with Low Attachment‐Produced Spheroids

**FIGURE 3:**
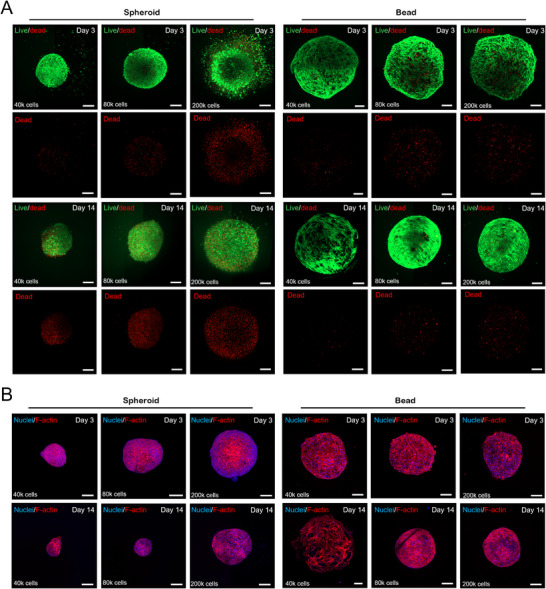
Viability and cell morphology analysis. (A) Live‐dead analysis of spheroids and living beads of corresponding cell number, at days 3 and 14 of maturation. (Green channel – Calcein AM; Red channel – Propidium Iodide). (B) F‐actin and nuclear staining of spheroids and living beads of corresponding cell number, at days 3 and 14 of maturation. (Blue channel – DAPI; Red Channel – Phalloidin). Scale bars: 200 µm.

Previous studies on the Cellgel technology have demonstrated that the metabolic glycoengineering can be successfully implemented across multiple cell types without compromising cell viability and metabolic activity [15]. To further validate our platforms as alternative living units to spheroids, the viability and cell morphology of both structures were studied through live/dead analysis and F‐actin staining, along the maturation time of 14 days. Live/dead analysis shows increased viability of living beads, especially at later time‐points (14 days), where increased cell death can only be observed for the spheroids (Figure 3A; Figure S1A) F‐actin staining demonstrates a more characteristic cell morphology of cells within living beads when compared to the corresponding spheroids, with adipose‐derived stem cells showing a spindle‐shaped morphology and cell spreading by day 3 in our platform, which is unattainable in standard spheroids until day 14 of culture (Figure 3B; Figure S1B). The presence of hyaluronic acid, an extracellular matrix element, within the living beads, likely provides anchorage points for the cells, with RHAMM and CD44 receptors most likely being involved in the connection between the cells and the tethering biopolymer [24, 25]. Together with the observed cell movement and migration within these systems [15], this enables the cells to spread toward their characteristic spindle‐like shape.

Cell‐ECM interactions widely affect and regulate cell behavior, including processes such as proliferation, migration, or differentiation [26]. Our results suggest that cells within the living beads have improved cell viability and characteristic cell morphology, which we hypothesized to be due to the initial presence of ECM components, even at low concentrations, that foster a more physiologically relevant 3D environment, with improved tissue‐like architecture and cell‐matrix interactions.

### 2.3 Establishment and Characterization of Endothelial and Breast Cancer Living Beads

**FIGURE 4:**
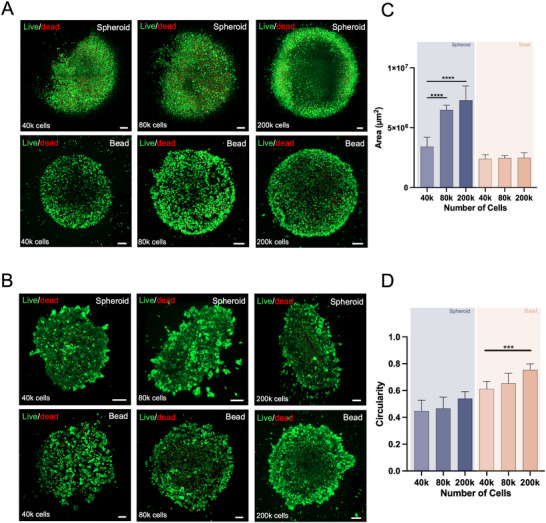
Development of living beads with other cell units. (A) Live‐dead analysis of spheroids and living beads of endothelial cells at day 3 of maturation. (Green channel – Calcein AM; Red channel – Propidium Iodide). Scale bars: 200 µm. (B) Live‐dead analysis of spheroids and living beads of breast cancer MCF‐7 cell line at day 3 of maturation. (Green channel – Calcein AM; Red channel – Propidium Iodide). Scale bars: 200 µm. (C) Endothelial spheroid and living bead area quantification, after 3.5 h of maturation and cross‐linking, respectively. Data presented as mean ± s.d. (*n* = 9, 3 technical replicates from three biological replicates). (D) Breast cancer spheroid and living bead circularity quantitative measurements, after 3.5 h of maturation and cross‐linking, respectively. (1 – perfect circle, 0 – line). Data presented as mean ± s.d. (*n* = 9, 3 technical replicates from 3 biological replicates).

To further investigate the potential of our methodology to be applied in diverse tissue engineering and disease modeling strategies, we established cell‐laden living beads using additional cell types and compared them to their corresponding spheroids. Living beads were successfully generated with endothelial (hUVECs) and breast cancer (MCF‐7 cell line) cells, with suitable cell viability (Figure 4A,B). Notably, the MCF‐7 breast cancer cell line is particularly challenging to self‐aggregate and form cohesive spheroids, when using ultralow attachment methods [27]. Therefore, by applying our methodology to this cell type, we aimed to improve the reproducibility of in vitro breast cancer models, thereby addressing a critical limitation in current spheroid formation methodologies, particularly with this specific cell line.

Our findings demonstrate that the bioorthogonal click chemistry methodology is versatile and applicable to several cell types. Identical to stem cells, it enabled the production of living beads with similar sizes across varying cell numbers, which was not achievable again with spheroids (Figure 4C; Figures S2 and S3) Notably, our cell‐laden living beads show improved circularity values compared to spheroids, even in cells that are less prone to aggregate (Figure 4D; Figure S2) Concerning the viability of these constructs, later time‐points, such as day 7 of maturation show the loss of viability within the spheroids, especially for endothelial cells, compared to the living beads. (Figures S2C and S3C) For breast cancer cell beads, lower seeding densities were associated with some cell loss, whereas beads formed at the highest density retained their structural integrity; notably, this condition most closely approximates the high cellularity observed in vivo. (Figure S3C) These findings demonstrate our methodology's capability to generate more uniform cell aggregates, which ultimately have an improved speed of production and develop more handleable structures than standard spheroid‐forming technologies.

Interestingly, when living beads were produced with the metastatic breast cancer cell line MDA‐MB‐231(M D Anderson‐Metastatic Breast‐231), the cells exhibited an invasive behavior toward a surrounding Matrigel matrix. Invasion was evident by day 3 of culture, and by day 14 of culture, no boundary between the initial living bead and the surrounding environment was distinguishable, with cancer cells fully disseminating throughout the entire structure. (Figure S4) These results indicate that our system preserves key morphological and biological properties of the embedded cells, enabling the production of models that retain relevant phenotypic features.

### 2.4 Establishment of Multi‐scale Human Living Materials Through Living Bead Units’ Fusion

**FIGURE 5:**
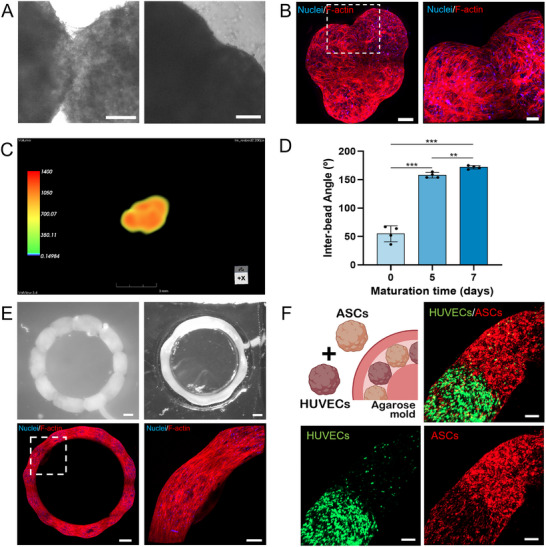
Establishment of Hierarchical Structures. (A) Optical microscope images of living beads at day 1 (left) and day 7 (right) of culture in contact. Scale bars: 200 µm. (B) F‐actin staining of whole fused construct (left) and close‐up of marked area (right) (Blue channel – DAPI; Red channel – Phalloidin). Scale bars: 200 µm (left), 100 µm (right). (C) 3D MRI image of living bead assembly in ultralow attachment plate. (D) Quantification of Inter‐bead Angle at days 0, 5, and 7 of maturation. Data presented as mean ± s.d. (*n* = 4, technical replicates). (E) Optical microscope images of living beads at day 1 and 7 of culture in contact (top left and right). F‐actin staining of whole fused construct (down left) and close‐up of marked area (down right) (Blue channel – DAPI; Red channel – Phalloidin). Scale bars: 500 µm (top; down left), 200 µm (down right). (F) Confocal microscope images of fused constructs comprised of hASC‐derived living beads, stained in red, and hUVEC‐derived living beads, stained in green. (Green channel – CMFDA; Red channel – DiD). Scale bars: 150 µm.

Spheroids, being cell‐rich structures, have the ability to fuse upon contact, creating larger unified structures [28]. This property is highly valuable in bottom‐up tissue engineering since it enables the creation of more complex assemblies, enhancing the biomimicry of such platforms [29]. Additionally, the fusion of smaller building blocks and development of structures with increasing complexity across different scales is important to improve mechanical integrity and functional integration [30]. In recent years, 3D bioprinting technologies enabled the successful printing and fusing of spheroids to create structures with increased complexity both in architecture and incorporated cell types [31]. Previous studies demonstrated that human‐cell‐based Cellgels, produced with this methodology, were able to fuse and self‐heal upon contact [15]. Building on this, we hypothesized that the living beads would be able to fuse and create more complex hierarchical structures, with diverse geometries. To test this, living beads were cultured in contact in ultralow attachment plates, for 7 days. Living beads image analysis reveal that the living units have well‐defined boundaries when placed together. Nevertheless, after 7 days in contact, the living beads form a seamless structure, that results from the fusion of the various building blocks (Figure 5A). F‐actin staining confirmed they were successfully fused and created seamless structures with different shapes (Figure 5B).

To further complement confocal imaging and assess the degree of fusion between the living beads, magnetic resonance imaging (MRI) was performed on a fused structure obtained through the culture of four living beads in contact, in an ultralow attachment plate well. The 3D reconstruction of the structure demonstrates the seamless fusion between its building blocks (Figure 5C). Interestingly, no distinct boundaries between the initial living beads were observed, displaying a homogenous pattern throughout the construct with minimal variations. (Video S1) These results suggest that the fusion process of living beads resulted in a cohesive and structurally uniform assembly.

To further understand the fusion process, the inter‐bead angles were measured between two adjacent living beads, cultured for 7 days (Figure S6). The angle between adjacent living beads increased over time of culture, from an average of 54.75°, at day 0, to 171.75°, at day 7 of culture, indicating a successful fusing process, that resulted in less pronounced edges between living beads and consequently increased angle values (Figure 5D).

To show that the living beads can be fused to more complex structures, living beads were cultured in contact within a circular agarose mold for 7 days (Figure S7). Both optical microscope and F‐actin staining images show that living beads were successfully fused (Figure 5E). Cellular bridging between living beads was observed, with cells extending across the junctions of the fused living beads, forming continuous and cohesive constructs (Figure 5E). Notably, we observed no apparent differences in fusion dynamics between the ultralow attachment plates and agarose molds, suggesting that the self‐assembly process is largely independent of the initial culture platform. The presence of F‐actin‐rich cellular bridges at living bead junctions further indicates active cytoskeletal remodeling during fusion, potentially contributing to tissue‐like mechanical properties. To investigate the influence of cell type composition in the fusion process, hierarchical structures with heterotypic living beads were developed and cultured in contact in the agarose molds. Results demonstrate that having living beads of different cell types did not prevent the fusing of these units (Figure 5F). In fact, not only did the fusing of the living beads occurred, but cells were also able to bridge and migrate toward the adjacent living beads, with increased migration from the stem cells, which are widely known to be highly mobile and to interact with endothelial cells to influence the overall cell's migration [5]. Interestingly, standard spheroids, which require longer aggregation time, show a lower circularity of each spheroid cultured together, after 4 h of aggregation. (Figure S5A) Additionally, it was possible to observe loose cells in the well, indicating that further aggregation time was necessary for the cells to aggregate, compared to our living bead units. After 7 days in culture together, the spheroids underwent extensive fusion, ultimately forming a single mass and losing their defined architecture. (Figure S5B,C)

### 2.5 Living Beads Retain Characteristic Cell Migration

**FIGURE 6:**
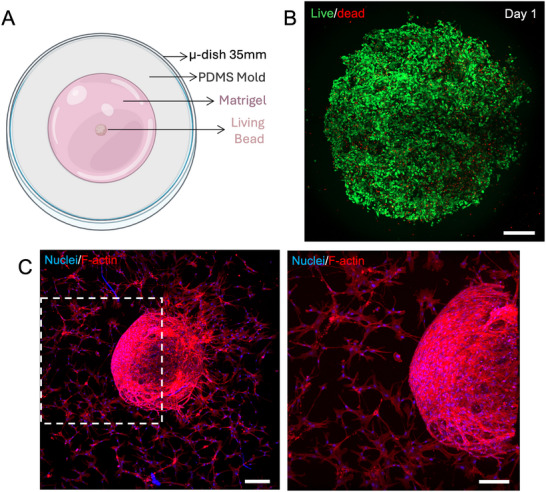
Living heterotypic living beads migration assay. (A) Schematics of the migration assay set‐up. (B) Live‐dead analysis of heterotypic living bead consisting of endothelial (hUVECs) and stem cells (hASCs) (1:3), at day 1 of culture. (Green channel – Calcein AM; Red channel – Propidium iodide). Scale bar: 300 µm. (C) F‐actin staining of the living bead and migrating cells at day 14 of culture within Matrigel. (Blue channel – DAPI; Red channel – Phalloidin). Scale bar: 300 µm. (left) Close‐up F‐actin staining of the marked area in the left. (Blue channel – DAPI; Red channel – Phalloidin). Scale bar: 200 µm. (right).

Cell migration is a key phenomenon for several pathological and physiological processes, in particular for tissue regeneration. Whereas several wound healing studies have been performed with 2D culture, by creating a cell‐free region in a cell monolayer that cells can bridge and repair, cells mostly migrate in a 3D state [32]. The 3D microenvironment plays a pivotal role in the migration behavior of cells, both through biochemical and biophysical cues. Cells within spheroids, such as mesenchymal stem cells and endothelial cells, have the freedom to migrate when in contact with other environments, namely with tissue‐mimetic and biocompatible hydrogels. This freedom to integrate their surrounding environments plays key roles in various biological scenarios, namely for effective tissue regeneration, as useful disease models portraying mechanisms such as metastasis and cancer progression, or even for processes such as angiogenesis [21, 33]. As such, the ability of cells within the living beads to migrate to surrounding tissues was evaluated. To do so, living beads composed of hUVECs and hASCs were produced in a ratio of 3:1, respectively, and placed within Matrigel, as a tissue biomimetic hydrogel (Figure 6A). The heterotypic living beads were developed as it is known that mesenchymal stem cells have a positive influence in endothelial cells, as supporting and vasculature inducing cells [34]. A live‐dead assay was performed to evaluate the viability of the two types of cells, and results showed that cells were viable after 1 day in culture, when they were transferred to Matrigel (Figure 6B). The living beads were left in culture for 14 days, and F‐actin staining results showed that cells within the living beads have the freedom to migrate toward the surrounding environment (Figure 6C,D). Interestingly, the cells that migrated show a spindle‐like shape, characteristic of the stem cells, indicating that most likely these cells have a higher migration rate and ability when compared to the endothelial cells. Indeed, studies show that mesenchymal stem cells have a high motility and migration capacity, particularly in ECM‐rich environments [35]. These characteristics are key in processes like tissue regeneration and angiogenesis. In the latter, hASCs play a crucial role in stimulating endothelial cells to form vascular‐like structures, a process that involves the tubulogenesis of endothelial cells, which most likely occurs closer to the cell aggregate [36].

### 2.6 Development of Modular Vascularized Human Living Materials

**FIGURE 7:**
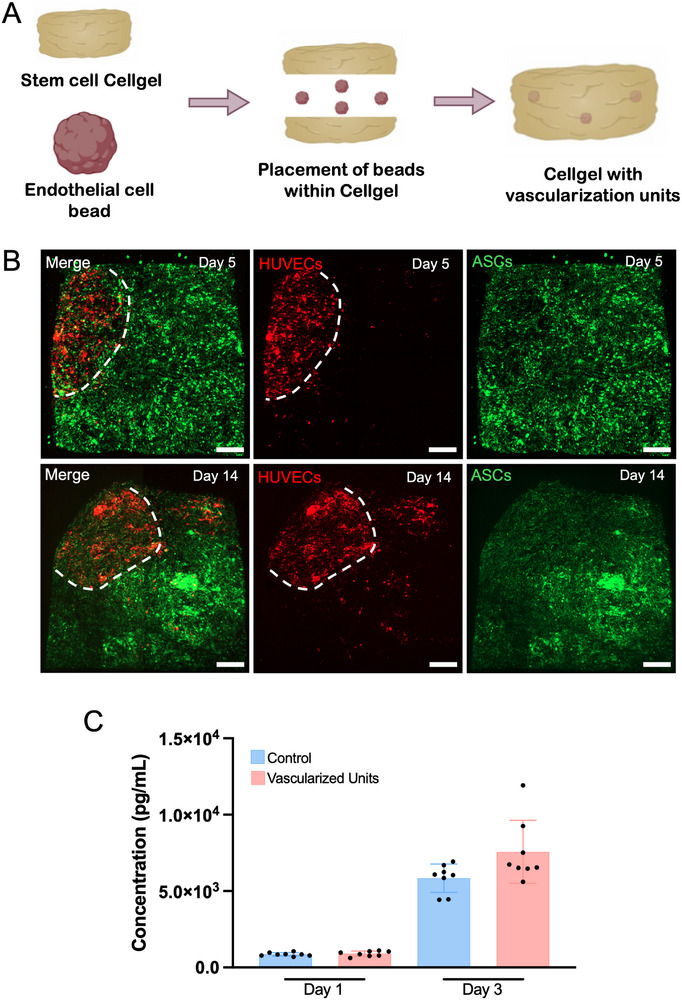
Generation of stem cell Cellgel with embedded vascular living bead units. (A) Schematic representation of the experimental set‐up for the vascularization unit Cellgels. (B) Confocal imaging of fluorescently labeled endothelial cell living beads within stem cell Cellgel, after 5 and 14 days of maturation. White dashed line corresponds to the living bead's boundary. (Red channel – DiD; Green channel – DiO). Scale bars: 200 µm. (C) ELISA quantification of VEGF release by control (Stem cell Cellgel) and the vascularized units (Stem cell Cellgel with embedded endothelial living beads), at day 1 and 3 of maturation. Data presented as mean ± s.d. (*n* = 8 technical replicates from 2 biological replicates).

Both in tissue engineering and disease modeling, the inclusion of vascular cell units is critical, as they are involved in several biological processes and in the successful integration of structures within tissues [37]. As such, the incorporation of endothelial living beads in mesenchymal stem cell‐derived Cellgels, cell‐rich platforms produced with the combination of metabolic glycoengineering of cells and the tethering hyaluronan biopolymer, was performed to demonstrate the possibility of creating vascularization units within tissue‐analogs (Figure 7A), that allow for the vascularization of such platforms when implanted. To visualize the integration of these living beads within the Cellgel and the endothelial cells’ behavior, hUVECs and hASCs were stained with lipophilic dyes, Vybrant DiD and DiO, respectively. Four living beads were placed within the Cellgel precursor solution, to be integrated inside the bigger platform. Confocal imaging of the vascularized units showed the successful integration of these living beads, in red, with the mesenchymal stem cell Cellgel, in green (Figure 7B). Additionally, it was possible to observe that not only were the living beads successfully integrated, but by day 5 there are cells migrating from the endothelial living bead toward their surroundings, with this migration being more prominent after 14 days of maturation of these structures.

Growth factors, namely vascular endothelial growth factor (VEGF), play a pivotal role in promoting angiogenesis, which is key for facilitating the integration of engineered tissues and biomaterials into host tissues [38]. Researchers have been developing materials with the controlled release of such molecules in order to achieve proper integration and regeneration of tissues [39]. Since with our methodology it was possible to integrate endothelial cell living beads within hASC‐derived Cellgels, we hypothesized that by combining both cell types, our vascularized unit‐including platforms would release pro‐angiogenic factors, providing beneficial characteristics for applications, such as tissue engineering. As such, an ELISA analysis of VEGF in days 1 and 3 of maturation of these structures was performed and compared to Cellgels without the living beads. At day 3 of maturation, although the vascularized constructs showed a slight increase when compared to control Cellgels, the difference was not statistically significant (Figure 7C). Mesenchymal stem cells are known for producing growth factors that enhance angiogenesis and promote endothelial cells’ survival and migration [40]. When in coculture, these cell types establish a paracrine effect, where endothelial cells upregulate factors that in turn promote the release of pro‐angiogenic factors by the mesenchymal stem cells [41]. This is upregulated when cocultured in 3D. Thus, despite the absence of a significant change in VEGF levels in our assays, it remains likely that additional angiogenic mediators most likely contribute to the overall pro‐angiogenic potential of the system. Key parameters such as the number of embedded vascular living bead units, or their cell densities can be easily tuned. Additionally, our methodology is highly modular and adaptable to different cell types, allowing its application across various fields, by adjusting cell types, densities and the structural composition of the platform or by providing dynamic culture conditions.

## 3 Conclusion

In this study, we explored a straightforward methodology to generate spherically‐shaped living bead units by using bioorthogonal click chemistry combined with in‐air fabrication in superhydrophobic surfaces, which is applied thereafter to generate multi‐scale human living materials. Compared to spheroids produced in low attachment plates, these units demonstrated improved cell viability and characteristic spindle‐like cell morphology, across maturation time. Interestingly, it was possible to produce living beads with various cell densities, but similar volumes, an advantage unattainable with current spheroid‐producing strategies. Living beads exhibited relevant features, including the capability of fusing to create multi‐scale living constructs, as well as the ability of migrating toward surrounding tissue‐like matrices. The modularity of such methodology enables both the production of bottom‐up structures for tissue engineering and their integration into other tissue engineering platforms, granting those with new functions or characteristics. Ultimately, this methodology offers improved scalability and living bead units’ fabrication time, providing a valuable platform with broad applicability for fields that highly benefit from living features.

## 4 Experimental Section/Methods

### 4.1 Cell Culture

All cells were manipulated under aseptic conditions and maintained in a temperature‐controlled environment at 37°C and a 5% CO_2_ atmosphere. Human adipose‐derived stem cells (hASCs) were obtained from subcutaneous adipose tissue and cultured in Minimum Essential Medium Eagle ‐ Alpha Modification (α‐MEM) medium, supplemented with sodium bicarbonate (2.2 g L^−1^), 10% (v/v) heat‐inactivated fetal bovine serum (FBS) and 1% antibiotic‐antimycotic mixture (streptomycin, amphotericin B and penicillin). Human umbilical vein endothelial cells (hUVECs) were isolated from the umbilical cord vein and were cultured in Medium 199 supplemented with sodium bicarbonate (2.2 g L^−1^), 20% (v/v) FBS, 1% antibiotic‐antimycotic mixture and 1% GlutaMAX. Additionally, the medium was freshly supplemented with heparin sodium salt (100 µg.mL^−1^) and endothelial cell growth supplement (40 µg.mL^−1^). The aforementioned tissues were obtained from Hospital do Baixo Vouga and Hospital da Luz, in Aveiro, after approval of the competent ethics committee and after informed consent was obtained from all participants. Luminal breast cancer MCF‐7 cell line (ATCC HTB‐22) was cultured in DMEM‐HG medium supplemented with sodium bicarbonate (2.2 g L^−1^), 10% (v/v) FBS and 1% antibiotic‐antimycotic mixture. MDA‐MB‐231‐Luc‐GFP (Green Fluorescent Protein) cell line (ATCC HTB‐26) as cultured in Roswell Park Memorial Institute (RPMI) medium supplemented with sodium bicarbonate (2.2 g L^−1^), 10% (v/v) FBS, and 1% antibiotic‐antimycotic mixture. Culture medium was exchanged every 3‐4 days and cells were subcultured using trypsin‐EDTA solution (0.25%).

### 4.2 Superhydrophobic Surfaces’ Production

Superhydrophobic surfaces were produced as previously described [20]. Briefly, 90 mm, non‐treated, petri dishes were evenly coated with FluoroThane‐MW reagent (WX 2100) and left to dry for 24 h, in a chemical fume hood. The surfaces were then intensively washed (ethanol, 70% v/v) and dried, at 50°C, for 24 h, in an oven.

### 4.3 Chemical Functionalization of HA‐DBCO

The functionalization of the hyaluronic acid with pedant DBCO moieties has been performed as previously described [15]. Briefly, the hyaluronic acid was first converted to the tetrabutylammonium salt, for solubilization in DMSO, followed by the chemical modification with DBCO‐PEG_4_‐NH_2_, using (Benzotriazol‐1‐yloxy)tris(dimethylamino)phosphonium hexafluorophosphate (BOP) as a coupling reagent. The HA‐DBCO product was then extensively dialyzed, followed by lyophilization and confirmation of successful modification by Proton Nuclear Magnetic Resonance (^1^H NMR) analysis. The final product yielded a final degree of substitution of 12%.

### 4.4 Assembly of Spheroids and Living Beads

Pristine cells were washed with dPBS, detached with trypsin‐EDTA, neutralized with their respective medium, and centrifuged at 300 g. The cells were then seeded in an ultralow attachment round‐bottom 96 well‐plate (4 × 10^4^, 8 × 10^4,^ and 2 × 10^5^ cells/well) and cultured under standard conditions, with medium exchange every 3 days.

The living beads were produced, as previously described [15]. hASCs and MCF‐7 cells were incubated with non‐natural mannosamine derivative bearing azide moieties (Ac4ManNAz) in standard medium, for 24 h. hUVECs were incubated for 48 h. After incubation, cells were washed with dPBS, detached with trypsin‐EDTA, neutralized with their respective medium, and passed through a cell strainer (100 µm), to remove cell aggregates. Cells were then centrifuged at 300 g and resuspended in HA‐DBCO (2% w/v), at three different cell densities (20, 40, and 100 × 10^6^ cells/cm^3^). Living beads were then produced using a mechanical electronic repeater pipette to dispense a fixed volume of 2 µL onto the superhydrophobic surface. The living beads were left to self‐assemble for 3.5 h, at 37°C and 5% CO_2_ atmosphere, after which they were transferred to an ultralow attachment round bottom 96 well‐plate and cultured under standard conditions, with medium exchange every 3 days.

### 4.5 Morphometric Characterization

Spheroid and living beads morphometric parameters (diameter, area, and circularity) were obtained using optical microscope images, after 4 h of self‐aggregation or formation, respectively. The images were analyzed using an open‐source software (ImageJ) and a supervised algorithm developed by Ivanov and colleagues [42].

### 4.6 Viability Analysis

To study the viability of the constructs, both were washed with dPBS and incubated for 30 min, in the cell incubator, with Calcein‐AM (1:200 dilution) and propidium iodide (1:200 dilution). After, the constructs were washed with dPBS and imaged in a confocal fluorescence microscope. The constructs’ viability was analyzed at different maturation time‐points (3, 7, and 14 days).

### 4.7 Morphological Analysis

Morphological analysis was performed through the labelling of filamentous actin structures. Briefly, the structures were fixed in formaldehyde (PFA, 4% v/v), for 30 min and washed twice with dPBS. They were then permeabilized with Triton X‐100 (0.5% v/v in dPBS) for 10 min, followed by the incubation with Flash Phalloidin Red 594 (1:1000 dilution) and a 10 min incubation with DAPI. The constructs were imaged in a confocal fluorescence microscope.

### 4.8 Migration Assay of Cancer Cell Beads

Living beads were produced with MDA‐MB‐231‐Luc‐GFP (40 × 10^6^ cells/cm^3^), as previously described. The living beads were cultured for 24 h, under standard conditions. A PDMS ring was placed in a µ‐Dish round imaging chamber (35 mm). Then, Matrigel growth factor reduced (4°C) was added to the previously chilled set‐up, and one living bead was placed in the middle. The set‐up was then placed inside the cell incubator, and the Matrigel was left to gel for 30 min, after which media was added to the µ‐Dish. The living beads were cultured for 14 days, with medium exchange every 2‐3 days. The constructs were imaged in a confocal fluorescence microscope.

### 4.9 Assembly of Hierarchical Constructs

Living beads of hASCs were assembled as previously described (40 × 10^6^ cells/cm^3^). Once they were fully crosslinked (3.5 h), the cellgel living beads were placed together in groups of either 4 or 5 living beads, in a single ultralow attachment plate well, and left to adhere for 7 days, in standard culture conditions. For spheroid fusion, hASCs were cultured in ultralow attachment plate wells and placed together after 4 h in culture. The spheroids were left to adhere for 7 days, in standard culture conditions. Alternatively, agarose molds were produced by pouring previously molten agarose (2% w/v dissolved in 0.9% w/v NaCl) onto resin molds, featuring cylinder‐shaped architectures, as shown in Figure S7. The produced molds were then sterilized and equilibrated in culture medium overnight, in the cell incubator. After 24 h of culture, the hASCs living beads (12) were placed together within the resultant cylinder‐shaped mold, and left to adhere for 7 days, in standard culture conditions. After the culture period, the hierarchical constructs were fixed in PFA (4% v/v), for 30 min, permeabilized with Triton X‐100 (0.5% v/v, in dPBS) for 10 min, followed by the incubation with Flash Phalloidin Red 594 (1:1000 dilution) and a 10 min incubation with DAPI. The constructs were imaged in a confocal fluorescence microscope.

For the heterotypic constructs, hUVECs were stained with CellTracker Green CMFDA, and hASCs were stained with Vybrant DiD dye. After trypsinization, cell pellets with 1 × 10^6^ cells/cm^3^ were resuspended in serum‐free culture medium containing CMFDA or DiD (5 µL of dye/mL of medium) and incubated in a water bath for 45 and 30 min (37°C, 50 rpm), respectively. Living beads were assembled as previously described (40 × 10^6^ cells/cm^3^). After 24 h of culture, the living beads (12) were placed together within the cylinder‐shaped mold, and left to adhere for 7 days, in standard culture conditions. After the culture period, the hierarchical constructs were fixed in PFA (4% v/v), for 30 min. The constructs were imaged in a confocal fluorescence microscope.

### 4.10 MRI Scanning of Hierarchical Structures

The living beads’ construct was assembled as previously described, placing 4 living beads in an ultralow attachment plate well, for 7 days. The construct was fixed in PFA (4% v/v), for 30 min, washed, and left in dPBS, until further analysis. The fused assembly was analyzed in a research MRI device (Pure Devices, 0.55 T) with a field homogeneity of 5 ppm within a 10 mm diameter Field of View (FoV), maximum gradients of 1 T m^−1^ (x,y) and 1.2 T m^−1^ (z). 3D images were acquired using a 3D spin echo sequence with a repetition time (TR) of 3 s and echo time (TE) of 3 ms. The image had 40 × 40 × 40 pixels and a dimension of 5 × 5 × 5 mm (voxel lateral size of 0.125 mm). After acquisition, the image was resized to 200 × 200 × 200 pixels. A region of interest (ROI) was selected using the Image Segmenter tool in MATLAB. Raw data acquisition was conducted in Matlab R2024, and the reconstructed image was processed in Matlab and in the VolView software (version 3.4, Kitware Inc., US).

### 4.11 Quantification of Inter‐Bead Angle

Optical microscope images of adjacent living beads were taken at days 0, 5 and 7 of maturation. Fusion angle was measured between adjacent living beads, as exemplified in Figure S6.

### 4.12 Migration Assay of Heterotypic Beads

Living beads were produced with hASCs and hUVECs (1:3, 40 × 10^6^ cells/cm^3^), as previously described. The heterotypic living beads were cultured for 24 h, under standard conditions. A PDMS ring was placed in a µ‐Dish round imaging chamber (35 mm). Then, Matrigel growth factor reduced (4°C) was added to the previously chilled set‐up and one living bead was placed in the middle. The set‐up was then placed inside the cell incubator and the Matrigel was left to gel for 30 min, after which a mixture of α‐MEM and M199 (1:3, 2 mL) media was added to the µ‐Dish. The living beads were cultured for 14 days, with medium exchange every 2‐3 days. The constructs were then fixed with PFA 4% v/v, for 1 h, at 37°C, followed by permeabilization with Triton X‐100 (0.5% v/v) and F‐actin staining with Flash Phalloidin Red 594 (1:1000 dilution) and nuclei staining with DAPI (10 min). The constructs were imaged in a confocal fluorescence microscope.

### 4.13 Assembly of Vascularized Constructs

After the glycoengineering process, hUVECs were stained with Vybrant DiD dye. After trypsinization, cell pellets with 1 × 10^6^ cells/cm^3^ were resuspended in 1 mL of serum‐free culture medium containing DiD (5 µL of dye/mL of medium) and incubated in a water bath for 30 min (37°C, 50 rpm). The cells were then centrifuged and resuspended in the HA‐DBCO precursor solution, and living beads were produced, as previously mentioned. The living beads were cultured for 24 h. After this period, hASCs were stained with Vybrant DiO dye, following the same procedure. The cells were resuspended in HA‐DBCO, and cylinder‐shaped Cellgels were manufactured in in‐house‐made PDMS molds (6 mm diameter × 2 mm height). Control Cellgels were produced with only hASC‐precursor solution, whereas the vascularized constructs were assembled by placing 4 hUVEC‐laden living beads within the hASCs Cellgel precursor solution. Both the control and vascularized constructs were crosslinked for 3.5 h and then were cultured in standard conditions. The migration of endothelial cells from the living beads was monitored through confocal fluorescence imaging.

### 4.14 VEGF ELISA Quantification

At the designated time points (days 1 and 3 of culture), media was collected and transferred into microtubes. Samples were then centrifuged at 1500 g for 5 min at 4°C, and the supernatant was stored at ‐20°C, until further analysis. On the day of quantification, samples were thawed at 4°C prior to processing. Cell‐mediated release of VEGF was confirmed with an ELISA MAX Deluxe Set Human VEGF (446504; BioLegend), performed according to the manufacturer's instructions.

### 4.15 Statistical Analysis

Data and statistical analysis were performed with GraphPad Prism 8 Software. One‐way ANOVA with Tukey post hoc test was used for statistical comparisons between groups (^*^ *p* < 0.05, ^**^ *p* < 0.01, ^***^ *p* < 0.001, ^****^ *p* < 0.0001). Comparisons found to be non‐significant were not marked on the graphs.

## Funding

European Research Council Advanced Grant REBORN (grant agreement number H2020‐ERC‐AdG–883370) and project CICECO‐Aveiro Institute of Materials, UIDB/50011/2020, UIDP/50011/2020, and LA/P/0006/2020, financed by national funds through FCT/MEC (PIDDAC).

## Conflicts of Interest

The authors declare no conflict of interest.

## Supporting information




**Supporting File**: smtd70469‐sup‐0001‐SuppMat.docx

**Supporting File**: smtd70469‐sup‐0002‐VideoS1.mp4

## Data Availability

The authors declare that all data supporting the findings of this study are included within the article and Supplementary Information files. Raw data is available upon request to the corresponding authors.
