# Supplementary material for: Rapid Generation of Fusable Cell Beads for Multi‐Scale Human Living Materials Assembly
Source: Small Methods. 2026 Jan 15;10(4):e01450. doi: 10.1002/smtd.202501450 (PMC12929925; doi:10.1002/smtd.202501450)
Supplement: Supplementary file 1 — Supporting File: smtd70469‐sup‐0001‐SuppMat.docx [file SMTD-10-e01450-s001.docx]

Supporting Information

Rapid Generation of Fusable Cell Beads for Multi-scale Human Living Materials Assembly

Beatriz S. Moura, Maria V. Monteiro, Joana F. Soeiro, Nuno J. O. Silva, Vítor M. Gaspar^*^, João F. Mano^*^

*
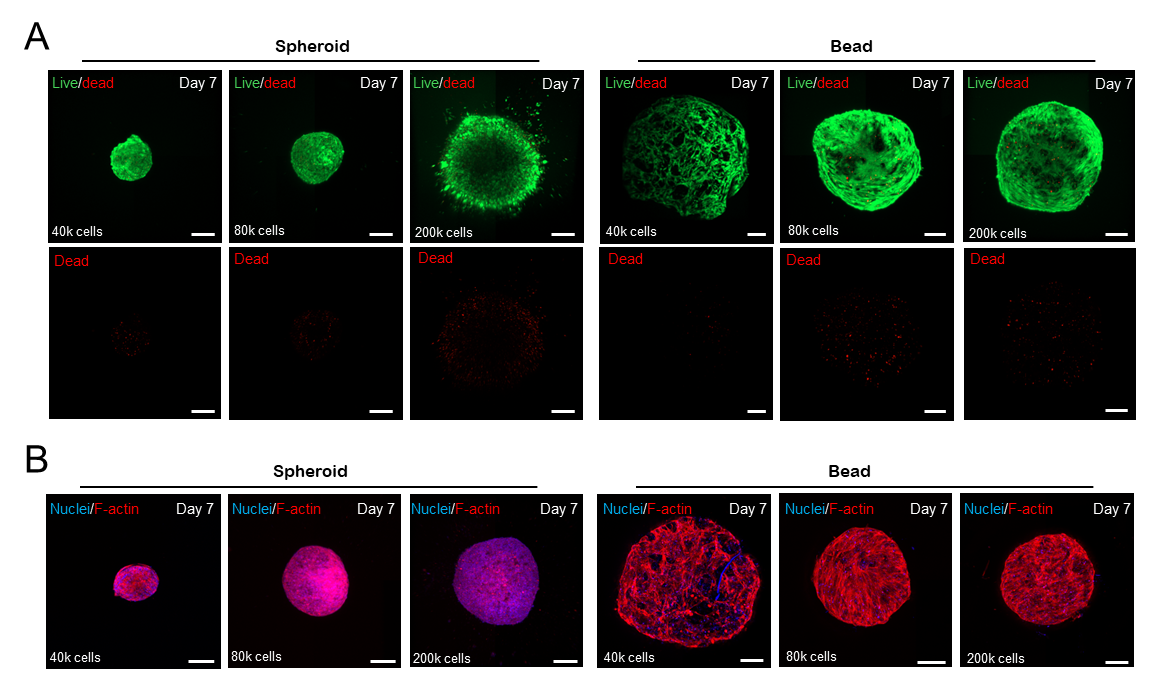
*

**Figure S1.** Viability and cell morphology analysis. A – Live dead analysis of spheroids and living beads of corresponding cell number, at day 7 of maturation. (Green channel – Calcein AM; Red channel – Propidium Iodide). B – F-actin and nuclear staining of spheroids and living beads of corresponding cell number, at day 7 of maturation. (Blue channel – DAPI; Red Channel – Phalloidin). Scale bars: 200µm

**
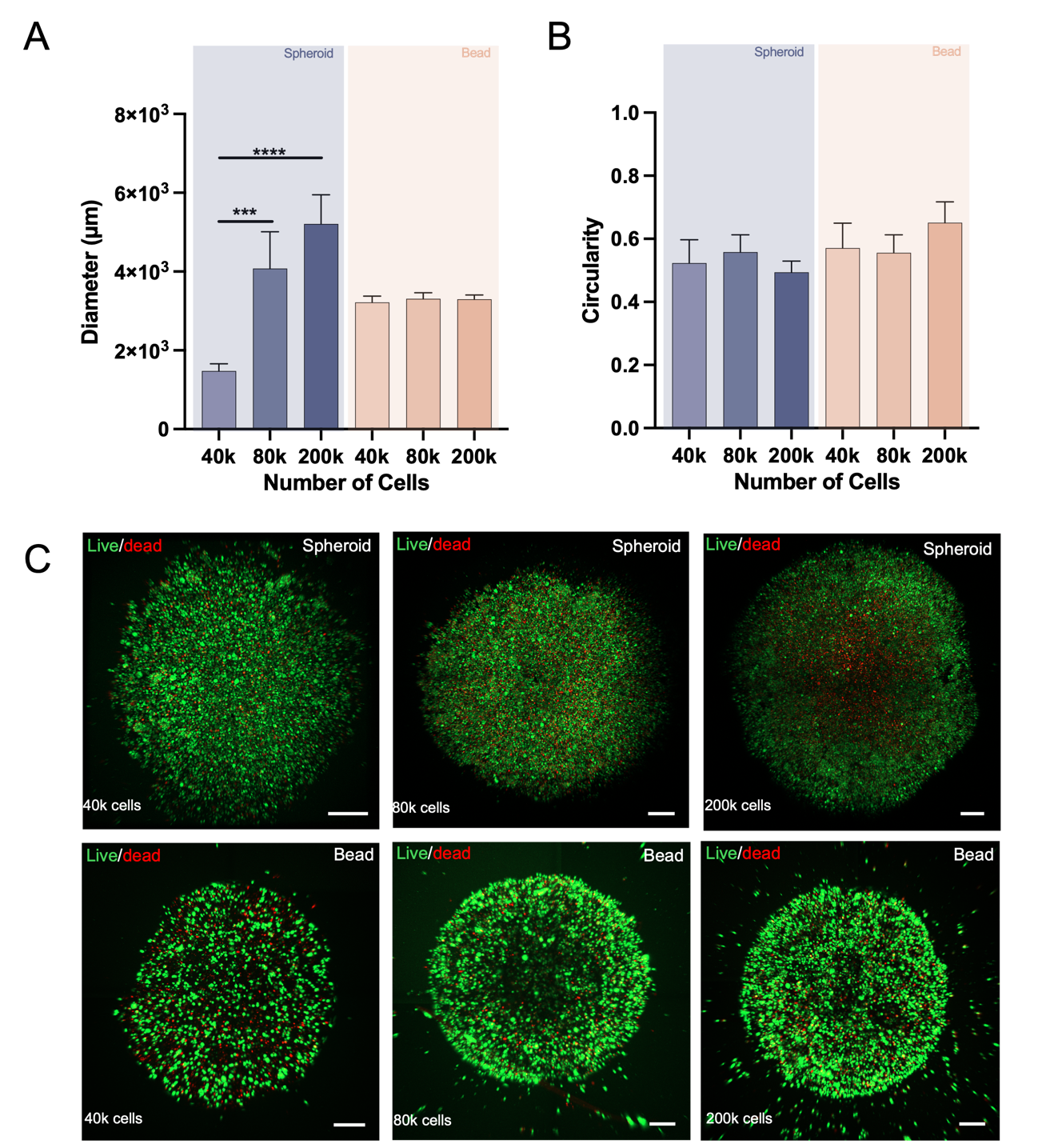
** **Figure S2.** A – hUVECs spheroid and bead diameter quantification, after 3.5h of maturation and cross-linking, respectively. Data presented as mean ± s.d. (n=9, 3 technical replicates from 3 biological replicates). B – hUVECs spheroid and bead circularity quantitative measurements, after 3.5h of maturation and cross-linking respectively. (1 – perfect circle, 0 – line). Data presented as mean ± s.d. (n=9). C – Live dead analysis of spheroids and living beads of endothelial cells at day 7 of maturation. (Green channel – Calcein AM; Red channel – Propidium Iodide). Scale bars: 200µm.


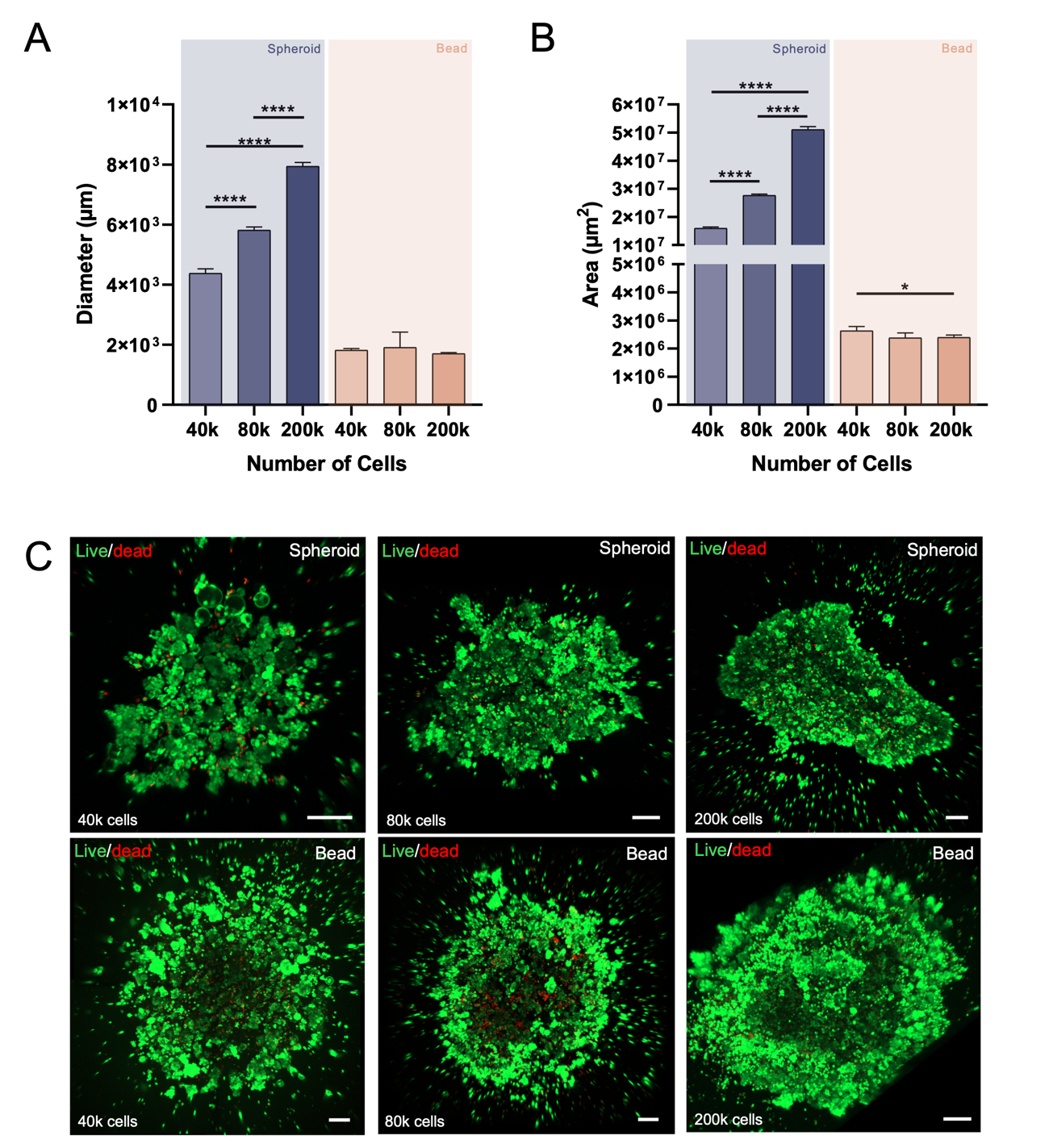


**Figure S3.** A – Breast cancer cell line MCF-7 spheroid and bead diameter quantification, after 3.5h of maturation and cross-linking, respectively. Data presented as mean ± s.d. (n=9, 3 technical replicates from 3 biological replicates). B – Breast cancer cell line MCF-7 spheroid and bead area quantification, after 3.5h of maturation and cross-linking respectively. Data presented as mean ± s.d. (n=9). C – Live dead analysis of spheroids and living beads of breast cancer cells at day 7 of maturation. (Green channel – Calcein AM; Red channel – Propidium Iodide). Scale bars: 200µm.


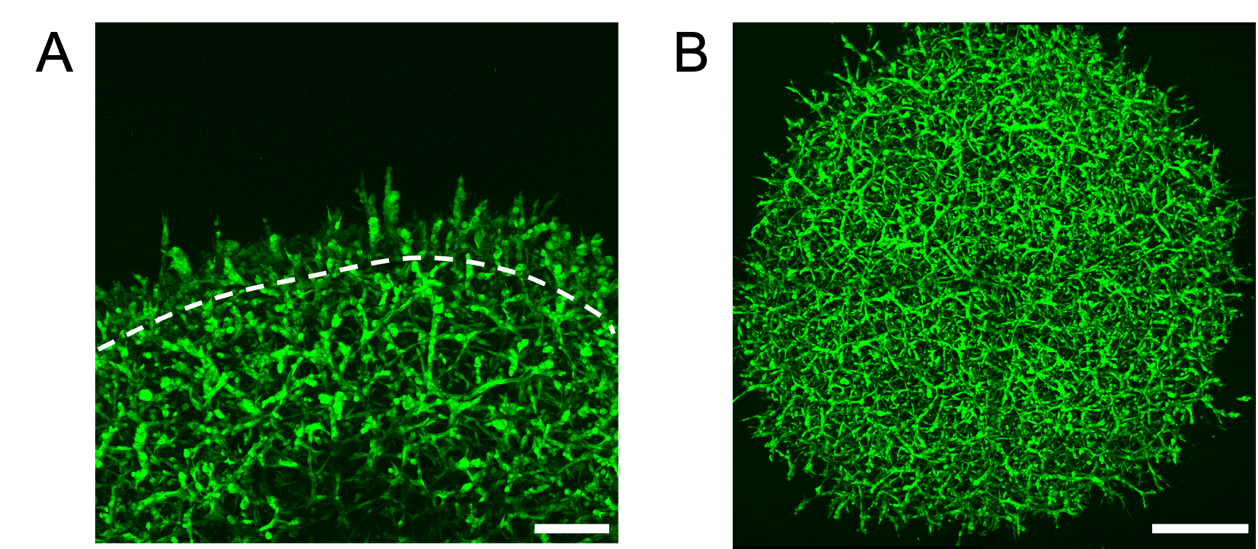


**Figure S4.** A – Breast cancer cell line MDA-MB-231 bead at day 3 of culture within Matrigel. (Green channel – GFP). Scale bar: 200um B – Breast cancer cell line MDA-MB-231 bead at day 14 of culture within Matrigel. (Green channel – MDA-MB-231 GFP cell line). Scale bar: 500μm


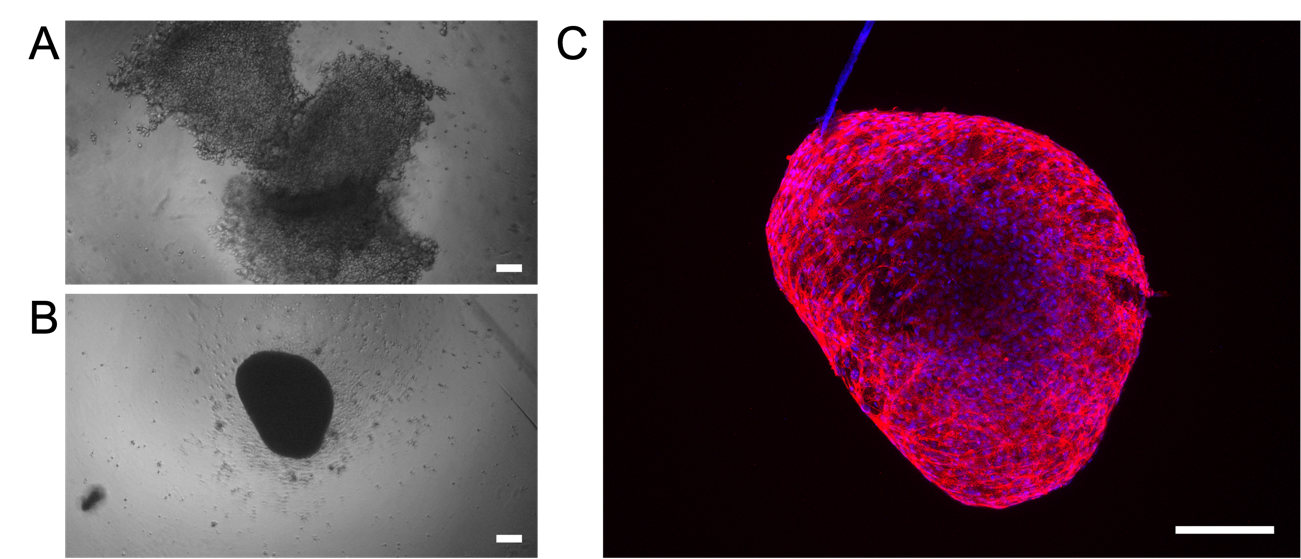


**Figure S5.** A and B – Optical microscope images of spheroids at day 1 (A) and day 7 (B) of culture in contact. Scale bars: 200µm. B – F-actin staining of whole fused construct. (Blue channel – DAPI; Red channel – Phalloidin) Scale bar: 200µm.


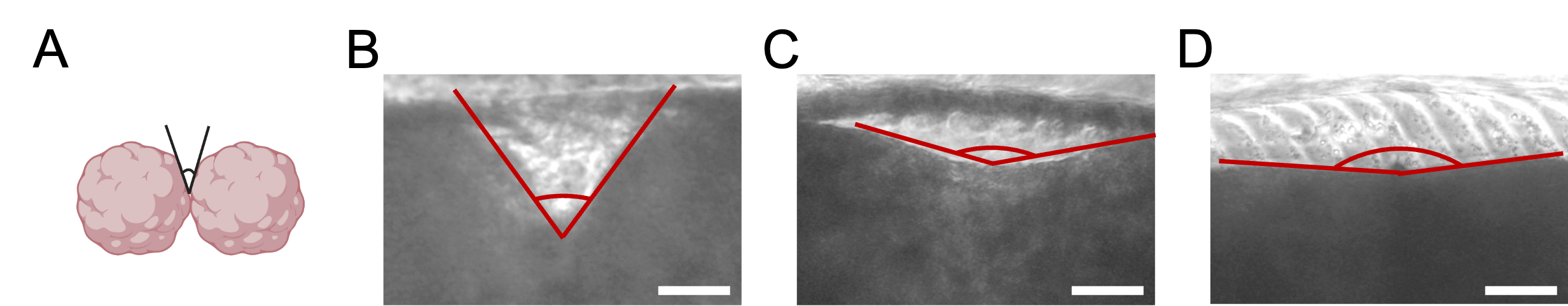


**Figure S6.** A – Schematic example of measured angle between fusing beads. B-D – Micrograph pictures of beads in culture for 0 (B), 5 (C) and 7 (D) days of maturation, and corresponding measured angle. Scale bars: 200µm


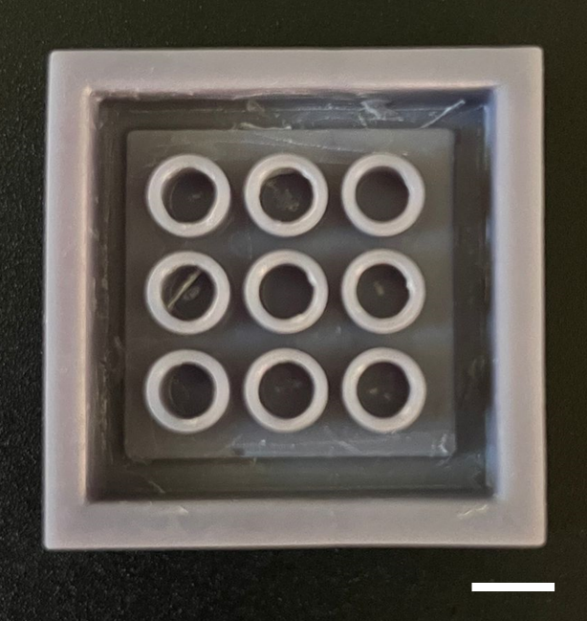


**Figure S7.** 3D printed resin negative mold for manufacturing the agarose mold. The resulting agarose mold have cilinders with an outer diameter of 5 mm, and inner diameter of 3 mm, resulting in a 1mm void space where the beads were cultured. Scale bar: 5 mm.
